# Effects of Berries, Phytochemicals, and Probiotics on Atherosclerosis through Gut Microbiota Modification: A Meta-Analysis of Animal Studies

**DOI:** 10.3390/ijms24043084

**Published:** 2023-02-04

**Authors:** Leila Khalili, Ann Marie Centner, Gloria Salazar

**Affiliations:** 1 Department of Nutrition and Integrative Physiology, College of Health and Human Sciences, Florida State University, Tallahassee, FL 32306, USA; 2 Center for Advancing Exercise and Nutrition Research on Aging (CAENRA), Florida State University, Tallahassee, FL 32306, USA

**Keywords:** berries, polyphenols, alkaloids, berberine, probiotics, atherosclerosis, *Akkermansia*, mouse, gut microbiota, meta-analysis

## Abstract

Atherosclerosis is a major cause of death and disability. The beneficial effects of phytochemicals and probiotics on atherosclerosis have gained significant interest since these functional foods can improve inflammation, oxidative stress, and microbiome dysbiosis. The direct effect of the microbiome in atherosclerosis, however, needs further elucidation. The objective of this work was to investigate the effects of polyphenols, alkaloids, and probiotics on atherosclerosis using a meta-analysis of studies with mouse models of atherosclerosis. Identification of eligible studies was conducted through searches on PubMed, Embase, Web of Science, and Science Direct until November 2022. The results showed that phytochemicals reduced atherosclerosis, which was significant in male mice, but not in females. Probiotics, on the other hand, showed significant reductions in plaque in both sexes. Berries and phytochemicals modulated gut microbial composition by reducing the Firmicutes/Bacteroidetes (F/B) ratio and by upregulating health-promoting bacteria, including *Akkermansia muciniphila*. This analysis suggests that phytochemicals and probiotics can reduce atherosclerosis in animal models, with a potentially greater effect on male animals. Thus, consumption of functional foods rich in phytochemicals as well as probiotics are viable interventions to improve gut health and reduce plaque burden in patients suffering from cardiovascular disease (CVD).

## 1. Introduction

Cardiovascular disease (CVD), in particular atherosclerosis, is one of the leading causes of mortality and remains a significant health burden [1]. Over 17 million people die from CVD worldwide, accounting for 31% of all deaths [2]. According to the World Health Organization (WHO), by 2030, over 23 million people will suffer from CVD worldwide [3].

Modifiable risk factors for atherosclerosis development include poor lifestyle choices such as high intake of foods rich is saturated fat and sugar and lower intake of foods rich in phytochemicals, such as fruits and vegetables. Poor lifestyle choices are associated with low grade inflammation, oxidative stress and elevated low-density lipoprotein (LDL) [4]. Dysbiosis of gut microbiota, characterized by reduced bacterial diversity and increased abundance of disease-promoting bacteria [5], is another important risk factor of atherosclerosis [6]. Gut dysbiosis is also associated with several human diseases, including obesity [7], type 2 diabetes [8], and hypercholesterolemia [9], all of which are risk factors for atherosclerosis development [10]. Furthermore, high fat diet (HFD)-induced microbiome dysbiosis correlates with plaque size and circulating cholesterol in Apolipoprotein E-deficient (ApoE^−/−^) mice [11]. Thus, the gut microbiota-derived metabolites have emerged as critical modulators of inflammation and lipid metabolism [10,12].

In the human microbiome, *Firmicutes* and *Bacteroidetes* are the most abundant phyla. The *Firmicutes*/*Bacteroidetes* (F/B) ratio has been used as a predictor of disease, as this ratio increases in obesity [13], with HFD in mouse models [14] and even during aging [15]. In contrast, this ratio decreases in certain conditions such as inflammatory bowel disease (IBD) [16]. Thus, improving microbiome health may reduce the risk of or improve CVD and has overall become an attractive target for therapeutic disease interventions, in particular through diet manipulations.

Bioactive compounds, including fiber and phytochemicals, are of particular interest as fiber is metabolized by the gut bacteria producing health-promoting short-chain fatty acids (SCFAs) such as butyrate, propionate, and acetate [17]. Phytochemicals, including polyphenols and alkaloids (berberine) have shown promising effects in reducing atherosclerosis and its risk factors. For example, polyphenols found in fruits, vegetables, nuts, teas, olive oil and spices improve microbiome health by diverse mechanisms including antibacterial activities against disease-promoting bacteria and improving healthy bacterial growth in the gut as well as prebiotic activities [18,19]. Berberine, an alkaloid found in medicinal plants and used for thousands of years in traditional Chinese medicine has shown potent effects against insulin resistance, oxidative stress, hypertension, hyperlipidemia, and inflammation [5,6,7].

Berries are good sources of phytochemicals, including polyphenols and alkaloids as well as fiber. We demonstrated that blackberry polyphenols reduced oxidative stress and senescence in vascular smooth muscle cells (VSMCs) in vitro [20] and that blackberry supplementation ameliorated atherosclerosis in ApoE^−/−^ male mice in vivo [21]. Females, however, were resistant to the plaque-lowering effects of this berry [21]. We also demonstrated that gallic acid, a polyphenol enriched in blackberry, mimicked the effect of this berry by reducing plaque in males, but not in female ApoE^−/−^ mice. The effect of gallic acid was associated with the restoration of *Eubacterium fissicatena* and *Turicibacter* levels and by the upregulation of *Akkermansia* in the gut [22]. Similarly, the reduction in plaque by berberine in ApoE^−/−^ male mice was also associated with the upregulation of *Akkermansia* [23], suggesting that this bacterium may be a common target of phytochemicals. These findings provide insights into the benefits of berry consumption in the improvement of CVD as berries are a rich source of phytochemicals that can partially restore microbiome dysbiosis and improve cardiometabolic health. 

Many studies assessing the effects of berries in atherosclerosis and the microbiome use only one sex; thus, the role of sex in microbiome modulation by diet is not fully understood. In light of this, we analyzed the results of studies evaluating the efficacy of polyphenols, alkaloid (berberine), and berries, as well as probiotics, that have direct effects on gut microbiota composition and plaque size in mice models of atherosclerosis. The findings revealed a significant sex-dependent effectiveness in plaque reductions. Phytochemicals were more effective in males, while probiotics reduced atherosclerosis in both sexes. Furthermore, increases in *Akkermansia muciniphila* abundance correlated with reduced plaque in several studies, suggesting that increasing the abundance of this strain in the gut is critical for the beneficial effects of phytochemicals in cardiovascular health.

## 2. Results

**Figure 1:**
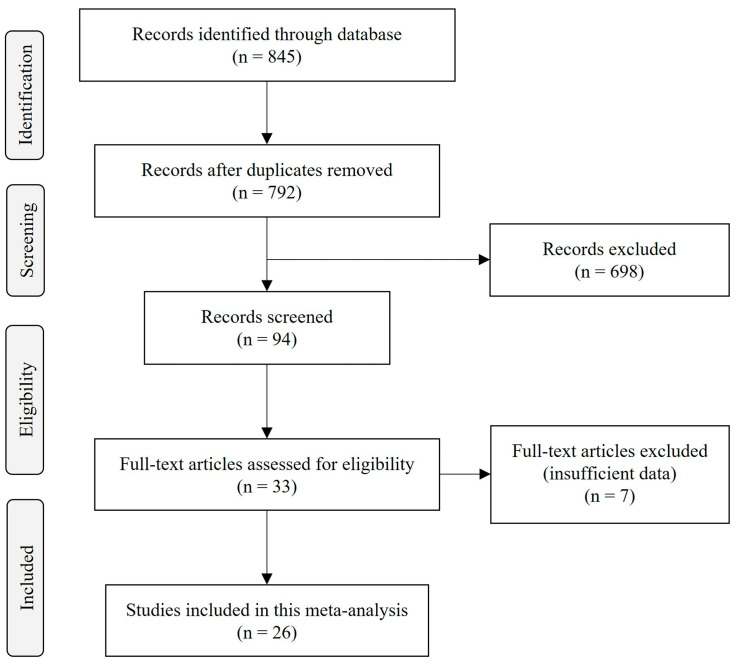
PRISMA flow diagram of the search strategy. Studies were identified through database searches in PubMed, Embase, Web of Science, and Science Direct until November 2022. The search included studies investigating the effects of berry, polyphenols, alkaloids, berberine and probiotics on gut microbiota and atherosclerosis plaque in ApoE^−/−^ or LDLR^−/−^ mice.

The initial search on diverse databases yielded 845 articles. After excluding duplicates, reviews, books, clinical trials, randomized control trials and meta-analyses, 94 articles were selected for potential inclusion. In total, 33 manuscripts contained original research testing the effect of polyphenols, berries, berberine or probiotics on microbiome composition and plaque size in mice. After reviewing the titles and abstracts, 7 studies were removed due to insufficient data and 26 studies were selected for the meta-analysis based on the inclusion criteria (Figure 1). 

For mouse models, inclusion criteria included ApoE^−/−^ and low-density lipoprotein receptor deficient (LDLR^−/−^) animals, of which ApoE^−/−^ is the most commonly used animal model of atherosclerosis. ApoE deficiency induces plaque accumulation over time, which is accelerated by HFD [24] and presents the most features of cardiometabolic syndrome [25,26]. 

### 2.1. Characteristics of Included Studies

The characteristics of the included studies are shown in Table 1. Of the 26 studies, the majority used ApoE^−/−^ mice (23 studies), and only three studies used LDLR^−/−^ mice. Considering sex, 9 studies used males, 10 used females, only 2 included males and females and 5 did not report the sex of the mice. Considering the type of intervention, 17 studies tested berries, polyphenols or berberine [22,23,27,28,29,30,31,32,33,34,35,36,37,38,39,40,41] and 9 used probiotics [11,42,43,44,45,46,47,48,49]. The intervention duration varied from 4 to 16 weeks of treatment, and all the studies used HFD to induce plaque. With respect to the microbiome, only 10 studies reported F/B ratios. For atherosclerosis quantification, all of the included studies, except for one [37], reported aortic plaque size, which was reported for the aortic root (7 studies) [32,33,36,40,47,48,49], the arch (4 studies) [34,35,42,46], the aortic sinus (11 studies) [11,28,29,30,31,38,40,41,43,44,45] and in the whole aorta (7 studies) [22,23,27,28,35,39,46]. The study by Yang et al. [37] reported a reduction in plaque in the aortic sinus by the intervention, but no quantification was provided. 

Considering probiotics, four studies used different strains of *Lactobacillus*, two studies used VSL#3, a probiotic mixture containing eight strains of bacteria (*Lactobacillus plantarum*, *Lactobacillus acidophilus*, *Lactobacillus casei*, and *Lactobacillus delbrueckii* subspecies *bulgaricus*, *Streptococcus salivarius* subspecies *thermophilus*, *Bifidobacterium infantis*, *Bifidobacterium longum*, and *Bifidobacterium breve*). Four studies used one of the following strains *Enterobacter aerogenes* ZDY01, *Lactobacillus reuteri DSM 1798*, *Pediococcus acidilactici* or *Akkermansia muciniphila*.

### 2.2. Berries, Polyphenols, and Alkaloids

#### 2.2.1. The Effect of Berries, Polyphenols, and Berberine in Plaque Burden

**Figure 2:**
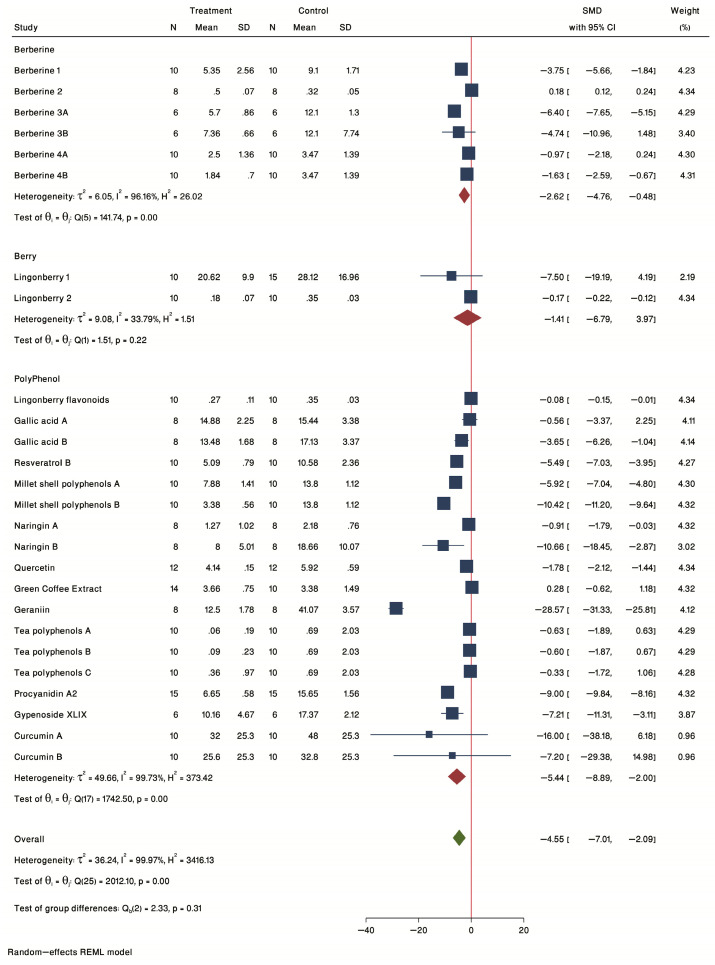
Forest plot of individual SMD of plaque size grouped by treatment type. Studies examining the effect of intervention in plaque size were divided in three groups including berberine, whole berries and probiotics. The common effect size was calculated as SMD and 95% CI for each study outcome. Squares represent findings for individual studies; red diamonds represent the overall result for each sub-group analysis and the green diamond the overall effect of the meta-analysis for all the studies.

A forest plot of individual effect sizes within each study for berries, polyphenols, and berberine in plaque burden is shown in Figure 2. The common standardized mean difference (SMD) from 17 studies in the 3 groups was −7.31 (95% confidence intervals (CI): −12.61 to −2.02, *p*-value < 0.05) based on a random effect model, with significant heterogeneity between studies (τ^2^ = 188.60, I^2^ = 99.99%, H^2^ = 17091.74, Q(df = 26) = 3374.41, PQ < 0.001). Further investigation by sensitivity analysis detected one study with a wide and unacceptable CI (SMD = −68.51, CI: −72.14 to −64.88) (Ming-liang Chen, 2016 A) that was removed from further analysis. The removal of this study resulted in a change in effect size (SMD = −4.55 (95% CI: −7.01 to −2.09), *p*-value < 0.05), with a slight decrease in heterogeneity, which remained significant (τ^2^ = 36.24, I^2^ = 99.97%, H^2^ = 3416.13, Q(df = 25) = 2012.10, PQ < 0.001). Considering all the studies, the effect of the overall interventions resulted in a significant reduction in plaque burden.

#### 2.2.2. Subgroup Analysis by Treatment Type

The forest plot of individual SMD of predetermined subgroup analysis by treatment type is presented in Figure 2. The results showed a non-significant treatment effect for the berry subgroup (SMD = −1.41, 95% CI = −6.79 to 3.97) that included 2 lingonberry studies, one in female [33] and the other in male [27] ApoE^−/−^ mice. A significant effect was seen for both polyphenols (SMD = −5.44, 95% CI = −8.89 to −2.00) and berberine (SMD = −2.62, 95% CI = −4.76 to −0.48) subgroups. The strongest reduction in plaque was seen for geraniin [32] in female ApoE^−/−^ mice. 

#### 2.2.3. Subgroup Analysis by Treatment Duration

**Figure 3:**
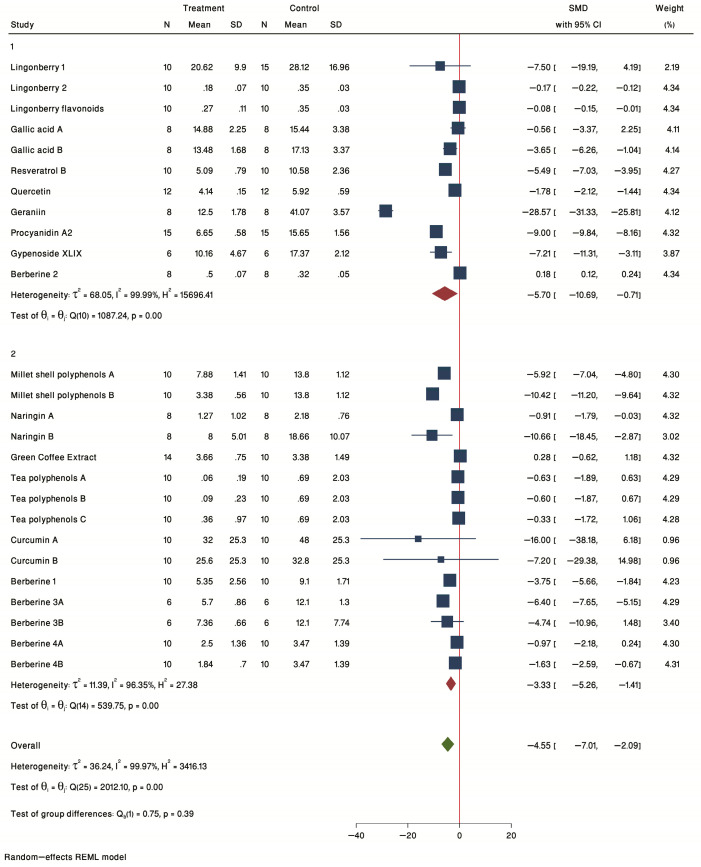
Forest plot of individual SMD of plaque size grouped by treatment duration. Nine studies were conducted for 4–12 weeks (1), and eight studies were conducted for more than 12 weeks (2). The common effect size was calculated as SMD and 95% CI for each study outcome. Squares represent findings for individual studies; red diamonds represent the overall result for each sub-group analysis and the green diamond the overall effect of the meta-analysis for all the studies.

The forest plot of individual SMD of predetermined subgroup analysis by treatment duration (1 = ≤12 weeks and 2 = >12 weeks) is presented in Figure 3. The results showed a significant treatment effect in both groups ≤12 weeks (SMD = −5.70, 95% CI = −10.69 to −0.71) and >12 weeks (SMD = −3.33, 95% CI = −5.26 to −1.41) subgroups. Thus, even 4 weeks of treatment is enough to see significant reductions in plaque, as seen by Ming-liang Chen, B [40] using 0.4% resveratrol in the diet in female ApoE^−/−^ mice. 

#### 2.2.4. Subgroup Analysis by Sex

**Figure 4:**
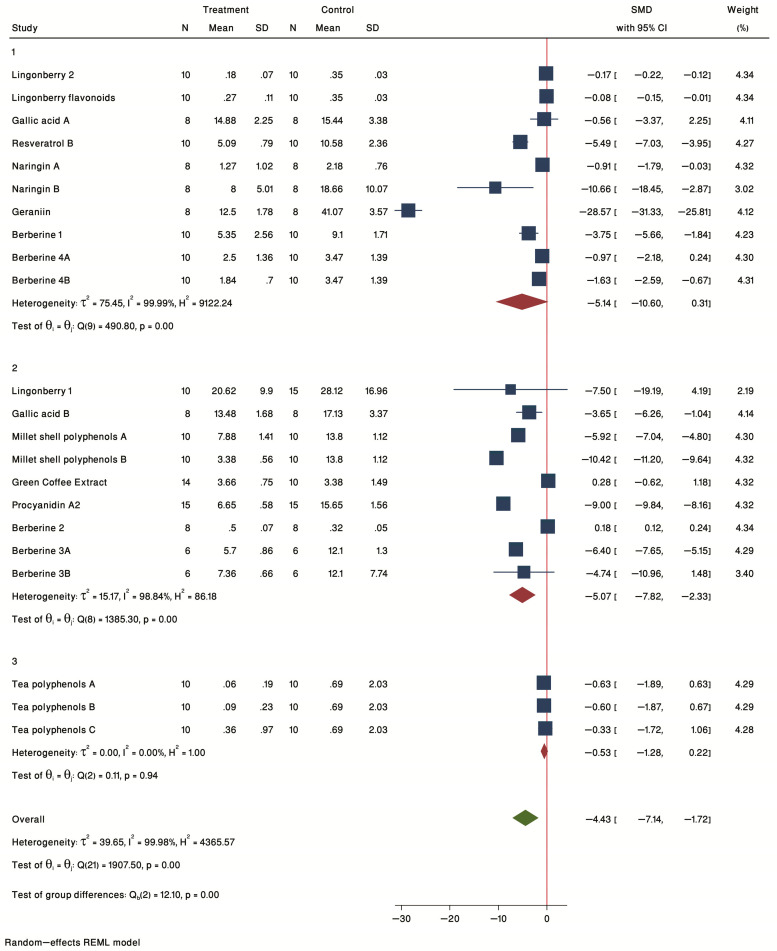
Forest plot of individual SMD of plaque size grouped by sex. The analysis included six studies conducted in female mice, six studies conducted in male mice, and two studies conducted in both sexes. The common effect size was calculated as SMD and 95% CI for each study outcome. Squares represent findings for individual studies; red diamonds represent the overall result for each sub-group analysis and the green diamond the overall effect of the meta-analysis for all the studies.

Next, we evaluated the effect of sex in plaque burden. The forest plot of individual SMD of predetermined subgroup analysis by sex (F = female, M = male) is presented in Figure 4. A significant treatment effect was seen for male (SMD = −5.07, 95% CI = −7.82 to −2.33), but not for female mice (SMD = −5.14, 95% CI = −10.60 to 0.31) or for studies using both sexes (M/F) (SMD = −0.53, 95% CI = −1.28 to 0.22). Thus, the overall significance seen for all studies was driven by the male studies. 

#### 2.2.5. Meta-Regression Results

Among our pre-specified potential moderators, sex of mice (male and female), treatment type (berry, polyphenol, berberine), treatment duration (≤12 weeks and >12 weeks), and study size did not significantly moderate the effect (*p* = 0.586, *p* = 0.728, *p* = 0.637, and *p* = 0.646, respectively). Therefore, these parameters were not a source of heterogeneity.

#### 2.2.6. Bias Assessment

**Figure 5:**
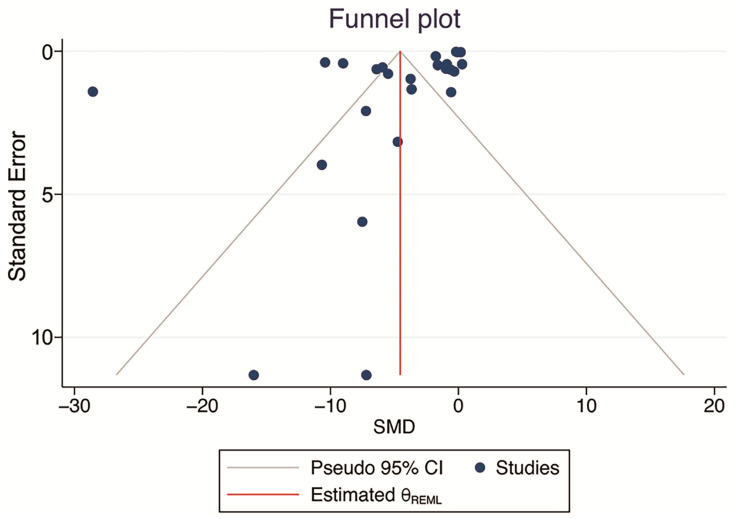
Funnel plot examining the publication bias of studies evaluating the effect of phytochemical supplementation on atherosclerosis plaque size. Meta-trim, a nonparametric “trim-and-fill” method, was used to estimate the number of possible studies missing from the meta-analysis because of publication bias. No missing studies were found.

There were no significant small-study effects according to Egger’s and Begg’s tests (*p* = 0.108, and *p* = 0.494; >0.05). Visual inspection of the funnel plot showed slight evidence of publication bias. However, the results of the analyses for the nonparametric “trim and fill” method showed no missing studies to be included, and the main SMD was retained (Figure 5). Therefore, there was no publication bias.

#### 2.2.7. Simple Correlation between Plaque and F/B Ratio

**Figure 6:**
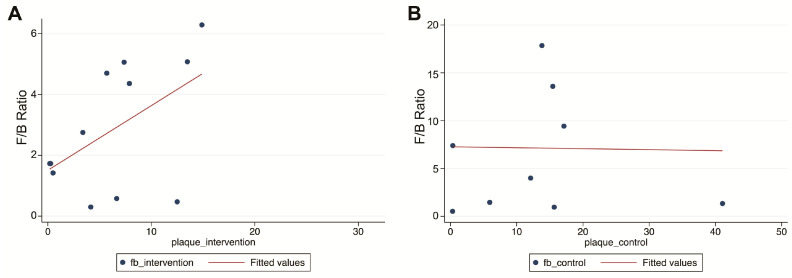
Scatterplot of the correlation between plaque size and F/B ratio. A positive correlation between plaque size (%) and F/B ratio was seen in the intervention groups (**A**), but not in the control groups (**B**).

From the eight studies reporting F/B ratios, a positive correlation between plaque and F/B ratio was seen; however, this correlation did not reach significance (r = 0.51, *p* = 0.087) (Figure 6A). Additionally, a negative correlation was seen for the control group, which was also non-significant (r = −0.01, *p* = 0.956) (Figure 6B).

### 2.3. Probiotics

#### 2.3.1. Aortic Plaque Size

**Figure 7:**
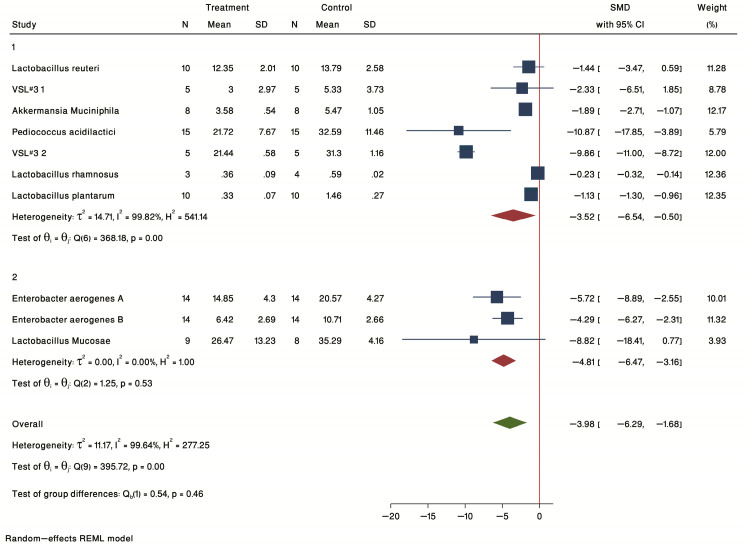
Forest plot of individual SMD of plaque size grouped by sex. Four studies were conducted in female mice, and 3 studies were conducted in male mice. The common effect size was calculated as SMD and 95% CI for each study outcome. Squares represent findings for individual studies; red diamonds represent the overall result for each sub-group analysis and the green diamond the overall effect of the meta-analysis for all the studies.

Nine studies treating ApoE^−/−^ mice with specific bacteria (Table 1, probiotics) showed a common SMD of −3.98 (95% CI: −6.29 to −1.68, *p*-value < 0.05) based on a random effect model, with significant heterogeneity between studies (τ^2^ = 11.17, I^2^ = 99.64%, H^2^ = 277.25, Q(df = 9) = 395.72, PQ < 0.001) (Figure 7). Sensitivity analysis detected no study with unacceptable CI. Thus, the forest plot of individual effect sizes within each study showed a significant reduction in plaque burden in the intervention group compared with controls. The most significant effects were seen for female ApoE^−/−^ mice treated with *Pediococcus acidilactici* [43], and VSL#3 [44] and for male ApoE^−/−^ mice treated with *Lactobacillus mucosae* [45].

#### 2.3.2. Subgroup Analysis by Treatment Duration

Probiotic studies were also analyzed by treatment duration for studies of ≤12 weeks (group 1) and >12 weeks (group 2) treatments (Figure 7). The results of the forest plot of individual SMD showed a significant treatment effect in both ≤12 weeks (SMD = −3.52, 95% CI = −6.54 to −0.50) and > 12 weeks (SMD = −4.81, 95% CI = −6.47 to −3.16) subgroups. The shortest duration tested in the included studies was for 8 weeks. This study treated male ApoE^−/−^ mice with *Akkermansia muciniphila* [47].

#### 2.3.3. Subgroup Analysis by Sex

**Figure 8:**
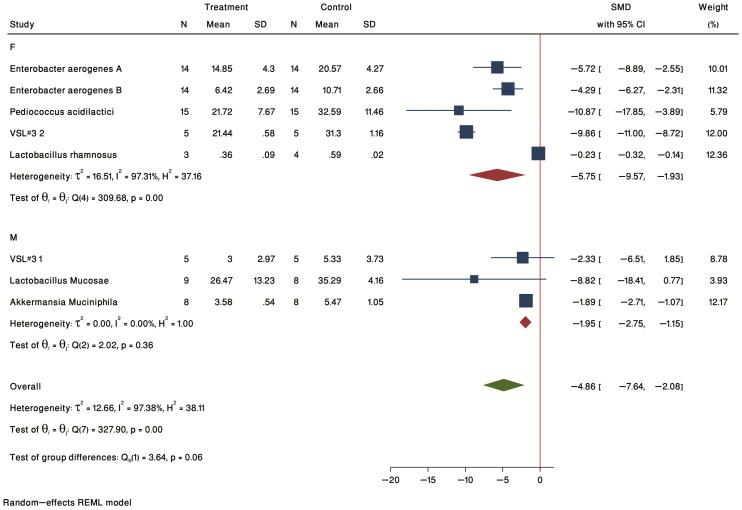
Forest plot of individual SMD of plaque size sub-grouped by sex. Four studies were conducted on females, and three studies were conducted on males. The common effect size was calculated as SMD and 95% CI for each study outcome. Squares represent findings for individual studies; red diamonds represent the overall result for each sub-group analysis and the green diamond the overall effect of the meta-analysis for all the studies.

In terms of sex (F = female, M = male, Figure 8), a significant treatment effect was seen for both male (SMD = −1.95, 95% CI = −2.75 to −1.15) and female (SMD = −5.75, 95% CI = −9.57 to −1.93) subgroups. 

#### 2.3.4. Meta-Regression 

Among sex of mice (male and female), duration of treatment (≤12 weeks and >12 weeks), and study size that could be potential moderators, none of these factors moderated the effect, significantly (*p* = 0.629, *p* = 0.744, and *p* = 0.489; respectively) so are not source of heterogeneity.

#### 2.3.5. Bias Assessment

**Figure 9:**
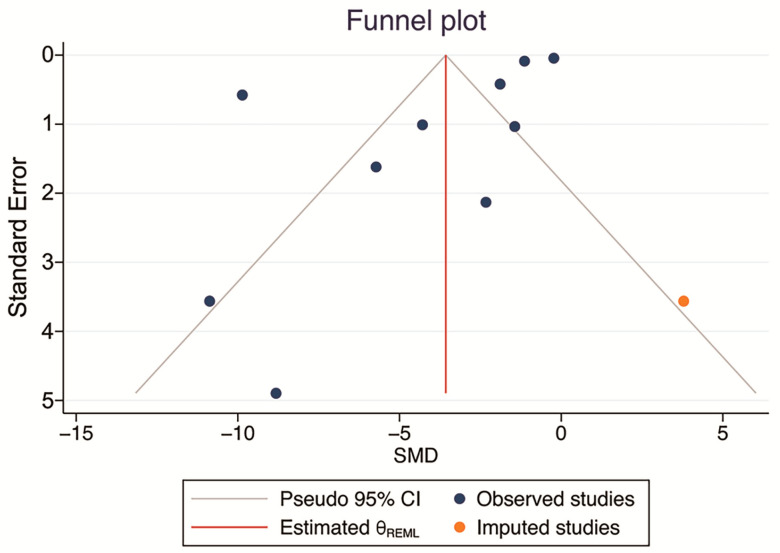
Funnel plot examining the publication bias of studies evaluating the effect of probiotics supplementation on atherosclerosis plaque size. Meta-trim, a nonparametric “trim-and-fill” method, was used to estimate the number of possible studies missing from the meta-analysis due to publication bias. One missing study was found.

Small-study bias, as measured by Egger’s and Begg’s tests, showed no significant results (*p* = 0.079, and *p* = 0.371; >0.05). However, visual assessment of the funnel plot showed slight evidence of publication bias. The nonparametric “trim and fill” results showed one missing study to be included. The re-estimation of the overall SMD after adding the “missing” studies, still resulted in a significant SMD (observed + imputed SMD = −3.56 (95% CI: −5.88 to −1.25)) (Figure 9).

## 3. Discussion

Functional foods, such as berries, have shown multiple health benefits in reducing CVD and its risk factors in both animal and clinical studies. Many of the benefits of these foods and their components (polyphenols, alkaloids, and fiber) are associated with improvements in microbiome heath, assessed, in part, by reductions in the F/B ratio. However, the direct effect of the microbiome on plaque burden needs further elucidation. The present meta-analysis analyzed animal studies that assessed the effects of berries (as whole food), polyphenols, berberine (as an alkaloid), and probiotics on atherosclerotic plaque size and microbiome modulation in mouse models of atherosclerosis (ApoE^−/−^ and LDLR^−/−^). The results showed that polyphenols, berberine, and probiotic interventions significantly reduced atherosclerotic plaque size in mice. The effect of berries was not statistically significant as there were limited studies that measured the effect of berries on the microbiome and atherosclerosis.

Polyphenols have shown promising effects in the treatment of chronic diseases as they exert powerful anti-inflammatory, anti-antioxidative, and cholesterol-lowering effects [50]. In human studies, a mixture of strawberries, bilberries, chokeberries, and black currants decreased systolic blood pressure and LDL, while upregulating HDL [51]. In another study, strawberry alone reduced LDL cholesterol as well as vascular cell adhesion molecule-1 (VCAM-1) levels in patients with metabolic syndrome [52]. In animal studies, blueberry [53], blackberry [21], and prunes [54] reduced plaque. Comprehensive reviews of nutritional interventions for CVD can be found in recent reviews [55,56,57]. However, only a limited number of studies in mice using berries as a whole food have measured plaque together with microbiome composition. We identified only two studies which used lingonberry as the intervention to reduce plaque. In one study by Matziouridou et al. [27], plaque size showed a positive correlation with *Bilophila*, *Mucispirillum*, *Turicibacter*, and *Lactococcus* and two unclassified bacterial genera in *Clostridiaceae* and *Peptostreptococcaceae*, while lingonberry increased the abundance of *Akkermansia muciniphila*, *Blautia producta*, *Clostridum difficile*, and *Eubacterium dolichum*. The second study by Liu et al. [31] showed a positive correlation between plaque and *Mucispirillum, Streptococcus, Peptococcaceae* and *Bilophila* genera. Similar to the previous study, lingonberry increased the abundance of *Akkermansia.* Thus, for these studies increases in *Mucispirillum* seems to be associated with plaque in both male and female mice, while *Akkermansia* was upregulated by lingonberry in both sexes. Major changes in the abundance of bacteria associated with interventions is shown in Table 2. 

The genus *Akkermansia*, which belongs to the *Verrucomicrobia* phylum, includes mucin-degrading bacteria, with the major species being *Akkermansia muciniphila*, a producer of acetate and propionate [58]. *Akkermansia muciniphila* abundance is upregulated with interventions improving metabolic disturbances. For example, its levels were elevated in obese mice treated with prebiotics, which correlated with improved metabolic status [59]. Upregulation of *Akkermansia* by metformin improved glucose homeostasis in diet-induced obesity in mice [60]. Additionally, the abundance of these bacteria is inversely associated with inflammatory diseases of the gut, such as IBD and Crohn’s disease [61]. 

Several of the studies we identified in this meta-analysis showed a negative correlation of *Akkermansia* abundance with plaque. For example, in our previous study using gallic acid, upregulation of *Akkermansia* correlated with a reduction in plaque in male mice. The lack of effect of gallic acid in females was associated with a reduction in *Akkermansia* [22]. Similarly, the plaque-reducing effects of resveratrol [40], quercetin [29], geraniin [32], procyanidinA2 [37], and berberine [23] were also associated with the upregulation of *Akkermansia* (Table 2). Another study using berberine [38] analyzing the microbiome at the phylum level reported an upregulation of *Verrucomicrobia*. Moreover, among these studies, resveratrol, geraniin and berberine supplementation [38] led to a reduction in TMAO levels. Consistent with the production of propionate, two studies using lingonberry in which *Akkermansia* was upregulated, reported increases in propionate [27,33].

Not all studies measured SCFAs to draw strong conclusions of the role the microbiome composition in the levels of SCFAs; however, these data suggest that *Akkermansia* reduces plaque burden, at least in part, by reducing the TMAO in circulation. Further evidence in favor of the protective effect of this genus in cardiovascular health was provided by Li et al. [47] using *Akkermansia muciniphila,* as a probiotic, to reduce plaque in male ApoE^−/−^ mice. Interestingly, the effects of *Akkermansia muciniphila* were associated with reduced endotoxemia-induced inflammation which was independent of changes in the lipid profile. More specifically, *Akkermansia muciniphila* reduced macrophage infiltration and the expression of intracellular adhesion molecule 1 (ICAM-1), monocyte chemoattractant protein 1 (MCP-1) and tumor necrosis alpha (TNFα) in the plaque. In circulation, MCP-1 and interleukin 1 beta (IL-1β) were also reduced by treatment. Thus, the anti-atherogenic effects of the nutritional interventions reviewed here are likely mediated by anti-inflammatory mechanisms driven by the microbiome, in particular by *Akkermansia muciniphila*.

The changes in *Akkermansia muciniphila* were observed in different sections of the gastrointestinal tract including the cecum [27,29,33], colon [32] and feces [22,23,37]. In terms of other similarities among studies, millet shell polyphenols [31] and quercetin [29] increased *Ruminococcus*, and resveratrol [40] and gypenoside [41] increased *Lactobacillus.*

Probiotics using different strains of *Lactobacillus* [11,42,45,49] reported reductions in plaque, except for *Lactobacillus reuteri* DSM 1798. Berberine [34] and *Enterobacter aerogenes* ZDY01 [46] increased *Turicibacter* abundance, while gallic acid [22] and *Lactobacillus Mucosae* [45] reduced its levels. *Blautia* was increased by gallic acid [27] and by berberine [34] in ApoE^−/−^ mice.

Among the probiotic studies, only four out of nine measured microbiome composition and only one measured TMAO. Several changes in the microbiome were observed. For example, the study by Hassan et al. [42] showed that treatment with *Lactobacillus plantarum* upregulated *Bacteroides*, *Bacteroidaceae*, *Parabacteroides* and *Tannerellaceae* and downregulated *Desulfovibrionaceae*, *Lachnospiraceae* and *Ruminococcaceae*, suggesting that changes in other bacteria, and not the probiotic itself, may mediate the reduction in plaque.

Alkaloids are naturally occurring compounds that have shown a wide range of protective effects, including reductions in inflammation, oxidative stress, and atherosclerosis [62]. For example, berberine, found in medicinal plants, has been used for centuries in the treatment of inflammatory diseases [63]. Similar to polyphenols, all berberine studies reduced plaque through modulation of gut microbiota composition and gut barrier function. Of the four berberine studies, three reported reductions in TMAO.

According to the results of the present study, the consumption of berries and polyphenols shows a positive correlation with the F/B ratio [22,29,31,32,33,37]; however, this correlation did not reach significance since not all studies reported this ratio. Only two berberine studies reported the F/B ratio, which was not improved by the intervention [34,38]. 

In terms of barrier function, only one study using curcumin reported a reduction in plasma LPS levels and improved intestinal barrier function [35]. Unfortunately, this study did not report specific changes in the microbiome. 

In terms of sex-dependent effects, only two studies used male and female mice, including our previous study testing gallic acid and the study by Liao et al. [39] testing tea polyphenols. As mentioned before, *Akkermansia* abundance showed a negative correlation with plaque since *Akkermansia* was increased in males (decreased plaque) and reduced in females (no effect on plaque) with gallic acid treatment. For the tea polyphenols study, only *Bifidobacterium* was measured. Thus, it is unknown if other bacteria genera were affected by treatment. However, overall sex difference analysis showed that berries, polyphenols, and berberine were more effective in male mice, while probiotic supplementation was effective in both sexes. Studies using both sexes are needed to evaluate the role of sex in the effects of the microbiome in atherosclerosis. It is possible that diet composition (fat and sugar in HFD), age, and hormone status may play a role in the effectiveness of dietary interventions in females. 

Duration sub-group analysis showed that both ≤12 weeks and >12 weeks interventions were effective. The minimum duration examined in the analyzed studies was 4 weeks for phytochemicals and 8 weeks for probiotics, suggesting that this time is sufficient to induce significant changes in the microbiome and plaque burden. 

The present study has some limitations. First, the number of studies evaluating the effect of whole berries supplementation on atherosclerosis plaque was low. Second, different types of polyphenol and probiotics supplementation have been used in different studies, although it is generally accepted that different types of polyphenols and probiotics have cardioprotective efficacy. Third, significant heterogeneity was found in most of the analyzed parameters, and the source of the heterogeneity was not explored further. Only random-effect models were used to address heterogeneity, which may have affected the strength and extrapolation of our conclusions. Forth, the F/B ratio was reported only in eight studies, which were conducted for different treatment durations. Fifth, there is insufficient data to evaluate the reasons female animals were not significantly affected by the supplementations that were effective in male animals. Additionally, only two studies used both sexes to assess the effect of the intervention. Further, in the probiotic studies, analyses of microbiome composition and gut-derived metabolites were needed to identify health-promoting bacteria. More studies are needed to identify the effective phytochemical types and probiotic strains as well as the effective dose to be proposed for therapeutic interventions. Finally, fecal transplantation studies are needed to demonstrate that microbiome modulation is the driver of the protective effects seen in the berry, polyphenols and berberine studies, as demonstrated by Li et al. [47]

In summary, this meta-analysis suggests that modulation of the microbiota by lingonberry, polyphenols, berberine and probiotics reduces plaque by a mechanism mediated, in part, by the upregulation of *Akkermansia* and a reduction in TMAO. We propose that a probiotic aimed to reduce plaque in patients suffering from cardiovascular disease should contain *Akkermansia muciniphila*, and other bacteria upregulated by different types of intervention, such as *Blautia* (lingonberry and berberine), *Turicibacter* (berberine and *Enterobacter aerogenes*) and different strains of *Lactobacillus* (*plantarum*, *rhamnosus* and *mucosae*).

## 4. Materials and Methods

### 4.1. Search Strategy

A search in databases including PubMed, Web of Science, Science Direct, and Embase was performed until November 2022 for studies investigating the effects of phytochemical and probiotic supplementation on gut microbiota and atherosclerosis plaque size in mice. The following search terms were used: (Berry OR Polyphenol OR Alkaloid OR Berberine OR Probiotic) AND (Atherosclerosis) AND (Plaque OR Lesion) AND (Gut microbiota OR Gut microbiome) AND (ApoE^−/−^ OR LDLR^−/−^) AND (Mice OR Mouse). Only studies published in English were included.

### 4.2. Inclusion and Exclusion Criteria

The inclusion criteria were (i) studies performed on mouse models of atherosclerosis and (ii) investigation of the effects of phytochemicals or probiotic supplementation on gut microbial composition and atherosclerotic plaque size. Exclusion criteria were (i) lack of sufficient information of findings and (ii) narrative review studies, meta-analyses, and any other type of article that is not an original research study.

### 4.3. Statistical Analyses

The analyses were performed using STATA17 (StataCorp, College Station, TX, USA). PRISMA was used for the reporting of presented studies, as this is the preferred platform for reporting data for systematic reviews and meta-analyses [64]. 

Random effect meta-analyses were performed using a restricted maximum likelihood approach [65]. As there might be unknown or unregistered studies that could not be accessed, the random-effect model was used. The heterogeneity between studies was evaluated using the Cochran I-Squared, Tau-squared, and Q tests. A considerable heterogeneity included values higher than 75% for I-squared [66]. The common effect size was reported as SMD (standardized mean difference) and 95% CI (confidence interval) for each study. 

Funnel plots and Egger’s [67] and Begg’s [68] tests were conducted to assess publication bias. Meta-trim was used in case of evidence for probable publication bias. This nonparametric “trim-and-fill” method estimates the number of potentially missing studies, imputes them, and re-computes the overall effect-size using the observed and imputed studies [67]. Furthermore, meta-regression analysis was performed to find the source of heterogeneity. Meta-regression investigates whether the moderators (sex, treatment type, treatment duration, and study size, in this study) can explain between-study heterogeneity. In addition, sensitivity analyses were conducted using the leave-one-out method, in which, one study is removed each time and the meta-analysis is carried out for the rest of the studies. 

The Pearson correlation coefficient was used to assess the correlation between plaque and the F/B ratio.

## 5. Conclusions

The present meta-analysis suggests that consuming polyphenols, berries (a rich source of polyphenols), berberine (as an alkaloid), and probiotics can improve atherosclerosis plaque size in mouse models of atherosclerosis. Moreover, this study revealed that in addition to their direct effect on cardiometabolic health, polyphenols, berries, and berberine could affect aortic plaque size through modulation of gut microbiota composition, as well as probiotic consumption. The other important finding of this meta-analysis was that the probiotic supplements reduce plaque in both sexes. In contrast, polyphenols, berries, and berberine showed plaque reducing effects mainly in male mice. In addition, *Akkermansia muciniphila* emerged as an important strain upregulated by several interventions. These findings may inform clinical trials using probiotics containing *Akkermansia muciniphila* to improve human health.

## Figures and Tables

**Table 1:** Characteristics of included studies.

N	ID		Genotype	Sex	Intervention	Dose	Method of Intervention	Duration (Week)	F/B		Analyzed Aortic Section
									Intervention	Control	
Berries, Polyphenols, and Alkaloids											
1	Chrysoula Matziouridou, 2016 [27]		ApoE^−/− 1^	M	Lingonberries	44%	In diet	8	-	-	whole aorta
2	Jiyun Liu, 2022 [33]	A	ApoE^−/−^	F	Lingonberry	63 g/kg	In diet	10.5	1.73	7.39	aortic root
		B	ApoE^−/−^	F	Lingonberry flavonoids	2 g/kg	In diet	10.5	1.73	7.39	aortic root
3	McKenzie Clark, 2022 [22]	A	ApoE^−/−^	F	Gallic acid	0.2%	In drinking water	7	6.29	13.59	whole aorta
		B	ApoE^−/−^	M	Gallic acid	0.2%	In drinking water	7	5.08	9.42	whole aorta
4	Ming-liang Chen, 2016 [40]	A	ApoE^−/−^	F	Resveratrol	0.4%	In diet	4	-	-	aortic root
		B	ApoE^−/−^	F	Resveratrol	0.4%	In diet	4	-	-	aortic sinus
5	Fengming Liu, 2021 [31]	A	ApoE^−/−^	M	Millet shell polyphenols	50 mg/kg	In diet	16	4.36	17.85	aortic sinus
		B	ApoE^−/−^	M	Millet shell polyphenols	100 mg/kg	In diet	16	2.75	17.85	aortic sinus
6	Feng Wang, 2020 [28]	A	ApoE^−/−^	F	Naringin	100 mg/kg	In diet	16	-	-	aortic sinus
		B	ApoE^−/−^	F	Naringin	100 mg/kg	In diet	16	-	-	whole aorta
7	J. Nie, 2019 [29]		LDLR^−/− 2^	-	Quercetin	100 µg/day	In diet	12	0.3	1.45	aortic sinus
8	Erika Caro-Gómez, 2019 [30]		ApoE^−/−^	M	Green coffee extract	220 mg/kg	Oral gavage	14	-	-	aortic sinus
9	Kaiyang Lin, 2022 [32]		ApoE^−/−^	F	Geraniin	80 mg/kg	In drinking water	12	0.47	1.33	aortic root
10	Zhen-Lin Liao, 2016 [39]	A	ApoE^−/−^	M/F	Tea polyphenols	1.6 g/L	In drinking water	16	-	-	whole aorta
		B	ApoE^−/−^	M/F	Tea polyphenols	0.8 g/L	In drinking water	16	-	-	whole aorta
		C	ApoE^−/−^	M/F	Tea polyphenols	0.4 g/L	In drinking water	16	-	-	whole aorta
11	Shiying Yang, 2021 [37]		ApoE^−/−^	M	Procyanidin A2	110 mg/kg	In drinking water	12	0.58	0.96	-
12	Ming Gao, 2022 [41]		ApoE^−/−^	-	Gypenoside XLIX	30 mg/kg	By gavage	6	-	-	aortic sinus
13	Siddhartha S. Ghosh, 2014 [35]	A	LDLR^−/−^	-	Curcumin	100 mg/kg	In drinking water	16	-	-	whole aorta
		B	LDLR^−/−^	-	Curcumin	100 mg/kg	In drinking water	16	-	-	aortic arch
14	Lin Zhu, 2018 [23]		ApoE^−/−^	F	Berberine	0.5 g/L	In drinking water	14	-	-	whole aorta
15	Yafei Shi, 2018 [38]		ApoE^−/−^	M	Berberine	50 mg/kg	Intragastric twice weekly	12	1.42	0.52	aortic sinus
16	Min Wu, 2020 [34]	A	ApoE^−/−^	M	Berberine	100 mg/kg	By gavage	13	4.7	4	aortic arch
		B	ApoE^−/−^	M	Berberine	50 mg/kg	By gavage	13	5.06	4	aortic arch
17	Xingxing Li, 2021 [36]	A	ApoE^−/−^	F	Berberine	100 mg/kg	By gavage	16	-	-	aortic root
		B	ApoE^−/−^	F	Berberine	200 mg/kg	By gavage	16	-	-	aortic root
Probiotics											
1	Jinghui Tang, 2021 [46]	A	ApoE^−/−^	F	*Enterobacter aerogenes* ZDY01	10^8^ cfu ^3^	In diet	16	-	-	aortic arch
		B	ApoE^−/−^	F	*Enterobacter aerogenes* ZDY01	10^8^ cfu	In diet	16	-	-	whole aorta
2	Frida Fak, 2012 [49]		ApoE^−/−^	-	*Lactobacillus reuteri* DSM 1798	10^9^ cfu	In drinking water	12	-	-	aortic root
3	Andrea Mencarelli, 2012 [48]		ApoE^−/−^	M	VSL#3	20 × 10^9^ cfu	In drinking water	12	-	-	aortic root
4	Tianyi Jiang, 2020 [45]		ApoE^−/−^	M	*Lactobacillus mucosae*	10^9^ cfu	By gavage	13	-	-	aortic sinus
5	Jin Li, 2016 [47]		ApoE^−/−^	M	*Akkermansia muciniphila*	5 × 10^9^ cfu	Oral gavage	8	-	-	aortic root
6	Taiji Mizoguchi, 2016 [43]		ApoE^−/−^	F	*Pediococcus acidilactici*	1.8 × 10^11^ cfu	In drinking water	12	-	-	aortic sinus
7	Yee Kwan Chan, 2016 1 [44]		ApoE^−/−^	F	VSL#3	2.78 × 10^11^ cfu	In diet	12	-	-	aortic sinus
8	Yee Kwan Chan, 2016 2 [11]		ApoE^−/−^	F	*Lactobacillus rhamnosus* GG	10^8^ cfu	In diet	12	-	-	aortic sinus
9	Adil Hassan, 2016 [42]		ApoE^−/−^	-	*Lactobacillus plantarum* ATCC 14917	10^9^ cfu	In diet	12	-	-	aortic arch

^1^ apolipoprotein E (Apoe) knockout; ^2^ LDL receptor knock-out; ^3^ colony forming unit.

**Table 2:** Summary of changes in the microbiome associated with interventions.

N	ID	Genotype	Sex	Intervention	Microbiome	Major Change in Bacteria by Intervention	Metabolites	Effect on Plaque
**Berries, Polyphenols, and Alkaloids**								
1	Chrysoula Matziouridou, 2016 [27]	ApoE^−/−^	M	lingonberries	Cecum	*Akkermansia muciniphila* , *Blautia producta* , *Clostridum difficile* , and *Eubacterium dolichum*	Propionate Acetate	Reduced
2	Jiyun Liu, 2022 [33]	ApoE^−/−^	F	Lingonberry	Cecum	*Akkermansia*	Propionate, TMAO	Reduced
3	McKenzie Clark, 2022 [22]	ApoE^−/−^	F	gallic acid	Feces	*Akkermansia and Dorea*	ND	No effect
		ApoE^−/−^	M	gallic acid	Feces	*Akkermansia* *Eubacterium fissicatena, Turicibacter and Dorea*	ND	Reduced
4	Ming-liang Chen, 2016 [40]	ApoE^−/−^	F	Resveratrol	Cecum	*Bacteroides* , *Lactobacillus*, *Bifidobacterium*, and *Akkermansia*	TMAO	Reduced
5	Fengming Liu, 2021 [31]	ApoE^−/−^	M	Millet shell polyphenols	Cecum	*Oscillospira* and *Ruminococcus* *Allobaculum*	ND	Reduced
6	Feng Wang, 2020 [28]	ApoE^−/−^	F	Naringin	Feces	*Eubacterium* *Bacteroides*, *Bifidobacterium*, *Clostridium*,	TMAO	Reduced
7	J. Nie, 2019 [29]	LDLR^−/−^	-	quercetin	Cecum	*Akkermansia* , *Bacteroides*, *Parabacteroides* and *Ruminococcus* *Lactobacillus*	Caecal bile acids	Reduced
8	Erika Caro-Gómez, 2019 [30]	ApoE^−/−^	M	Green Coffee Extract	Feces	*Desulfovibrio and Mogibacteriaceae*	ND	No effect
9	Kaiyang Lin, 2022 [32]	ApoE^−/−^	F	geraniin	Colon	*Bacteroides* , *Alloprevotella*, *Alistipes,* and *Akkermansia*	TMAO	Reduced
10	Zhen-Lin Liao, 2016 [39]	ApoE^−/−^	M/F	Tea polyphenols	Feces	*Bifidobacterium*	ND	Reduced
11	Shiying Yang, 2021 [37]	ApoE^−/−^	M	Procyanidin A2	Feces	*Akkermansia, unclassified _f_Prevotellaceae and Coriobacteriaceae_UCG_002*	ND	Reduced
12	Ming Gao, 2022 [41]	ApoE^−/−^	-	Gypenoside XLIX	Feces	*Eubacterium, Roseburia, Bifidobacterium, Lactobacillus, and Prevotella* *Clostridioides and Desulfovibrionaceae*	TMAO Butyrate Propionate Acetate	Reduced
13	Siddhartha S. Ghosh, 2014 [35]	LDLR^−/−^	-	Curcumin	-	ND	LPS	Reduced
14	Lin Zhu, 2018 [23]	ApoE^−/−^	F	Berberine	Feces	*Akkermansia, Bacteroides* Desulfovibrio spp	ND	Reduced
15	Yafei Shi, 2018 [38]	ApoE^−/−^	M	Berberine	Feces	*Verrucomicrobia* *Proteobacteria*	TMAO	Reduced
16	Min Wu, 2020 [34]	ApoE^−/−^	M	Berberine	Feces	*Turicibacter* and *Alistipes* (High dose) *Turicibacter*, *Allobaculum* and *Blautia* (low dose)	ND	Reduced
17	Xingxing Li, 2021 [36]	ApoE^−/−^	F	Berberine	Cecum	*Lachnospiraceae* NK4A136, *Bacteroidales* S24-7 (unclassified), *Eubacterium*, *Marvinbryantia*, *Clostridiales* unclassified, *Ruminiclostridium 5*, *PrevotellaceaeNK3B31, Bifidobacterium*	TMAO	Reduced
**Probiotics**								
1	Jinghui Tang, 2021 [46]	ApoE^−/−^	F	*Enterobacter aerogenes* ZDY01	Cecum	*Turicibacter* and *unidentified_Ruminococcaceae*	TMAO	Reduced
2	Frida Fa°k, 2012 [49]	ApoE^−/−^	-	*Lactobacillus reuteri* DSM 1798	-	ND	ND	No effect
3	Andrea Mencarelli, 2012 [48]	ApoE^−/−^	M	VSL#3	-	ND	ND	Reduced
4	Tianyi Jiang, 2020 [45]	ApoE^−/−^	M	*Lactobacillus mucosae*	Feces	*Oscillibacter, Ruminiclostridium* , *Harryflintia*, *Enterorhabdus*, *Anaerovorax*, *Eubacterium*, *Turicibacter*, *Enterococcus*, unclassified *Ruminococcaceae*, unclassified *Clostridiales*, unclassified *Lachnospiraceae*	ND	Reduced
5	Jin Li, 2016 [47]	ApoE^−/−^	M	*Akkermansia muciniphila*	-	ND	ND	Reduced
6	Taiji Mizoguchi, 2016 [43]	ApoE^−/−^	F	*Pediococcus acidilactici*	-	ND	ND	Reduced
7	Yee Kwan Chan, 2016 (1) [44]	ApoE^−/−^	F	VSL#3	-	ND	ND	Reduced
8	Yee Kwan Chan, 2016 (2) [11]	ApoE^−/−^	F	*Lactobacillus rhamnosus* GG	Colon	*Lactobacillus* and *Clostridum*	ND	Reduced
9	Adil Hassan, 2016 [42]	ApoE^−/−^	-	*Lactobacillus plantarum* ATCC 14917	Feces	*Bacteroides* , *Bacteroidaceae*, *Parabacteroides* and *Tannerellaceae* *Desulfovibrionaceae, Lachnospiraceae and Ruminococcaceae*	ND	Reduced

N: refers to the number of the study in Table 1; ND: not determined; garnet color indicates increase and blue color indicates reduction.
